# Complete Mitochondrial Genome Sequence of an Australian Little Crow (Corvus bennetti)

**DOI:** 10.1128/mra.01367-22

**Published:** 2023-04-04

**Authors:** Subir Sarker

**Affiliations:** a Department of Microbiology, Anatomy, Physiology and Pharmacology, School of Agriculture, Biomedicine and Environment, La Trobe University, Melbourne, Victoria, Australia; Vanderbilt University

## Abstract

This study reports the complete mitochondrial genome sequence of an Australian little crow (Corvus bennetti). The circular genome has a size of 16,895 bp and contains 13 protein-coding genes, 22 tRNA genes, and two rRNA genes. The study provides a reference mitochondrial genome of a little crow for further molecular studies.

## ANNOUNCEMENT

Within the family Corvidae, crows (genus *Corvus*) make up about one-third of the species diversity (40 species), and they occur on all continents except South America and Antarctica, as well as in remote archipelagos, such as Hawaii, Micronesia, and Melanesia (1). The little crow (Corvus bennetti) is an Australian species of crow, very similar to the Torresian crow in having white bases to the neck and head feathers, but slightly smaller and with a slightly smaller bill. Molecular studies of *C. bennetti* have been based on only partial mitochondrial or nuclear gene sequences (2, 3). Therefore, the present study was designed to sequence a mitochondrial genome of *C. bennetti*.

A cutaneous tissue sample was collected from an Australian little crow (*C. bennetti*) that was euthanized due to injury by a registered veterinary doctor at Burwood Bird and Animal Hospital (Burwood, Victoria). Animal sampling (sample ID 122740) was carried out by the attending veterinarian, and the specimen was deposited at La Trobe University. The animal was necropsied by a registered veterinarian for routine diagnostic purpose. All other methods were performed in accordance with the standard guidelines and regulations for PC2 laboratory. The Animal Ethics Committee at La Trobe University was informed that findings from the diagnostic material were to be used in a publication, and a formal waiver of ethics approval was granted. For DNA extraction, the skin tissue material was aseptically dissected, mechanically homogenized in lysis buffer using disposable tissue grinder pestles, and transferred into a 1.5-mL microcentrifuge tube (Eppendorf). Total genomic DNA (gDNA) was extracted using a ReliaPrep gDNA tissue miniprep system (Promega, USA) according to the manufacturer’s guidelines, with minor modifications as per the stated protocols (4, 5). A total of 250 ng of extracted genomic DNA was used to prepare a DNA library using the protocol adapted previously with the Illumina DNA prep kit (San Diego, CA, USA) (6), and the library was sequenced using the Illumina NovaSeq sequencing platform, generating 150-bp paired-end reads (7, 8). The sequencing data were analyzed as per the established pipeline (7–10) using Geneious Prime version 2022.1.1 (Biomatters, New Zealand) and CLC Genomics Workbench version 9.0.1. Briefly, a total of 39.8 million raw reads were preprocessed to remove the Illumina adapters, ambiguous base calls, and poor-quality reads (trimmed using a quality score limit of 0.05; ambiguous reads of up to 15 nucleotides trimmed using CLC Genomics Workbench), followed by mapping the reads against an Escherichia coli bacterial genomic sequence (GenBank accession number U00096) to eliminate possible bacterial contamination. A total of 30.2 million trimmed and unmapped clean reads were used as input data for *de novo* assembly using CLC Genomics Workbench with default parameters. This resulted in the generation of a 16,895-bp mitogenome obtained from *C. bennetti* with an average coverage of 47.99×. The final mitogenome of *C. bennetti* was circularized using Geneious software with default parameters. Annotation of the assembled mitogenome of *C. bennetti* was performed with default parameters using the vertebrate mitochondrial genetic code (transl_table 2) using Geneious Prime version 22.1.1. All software was used with default parameters except where stated.

**FIG 1:**
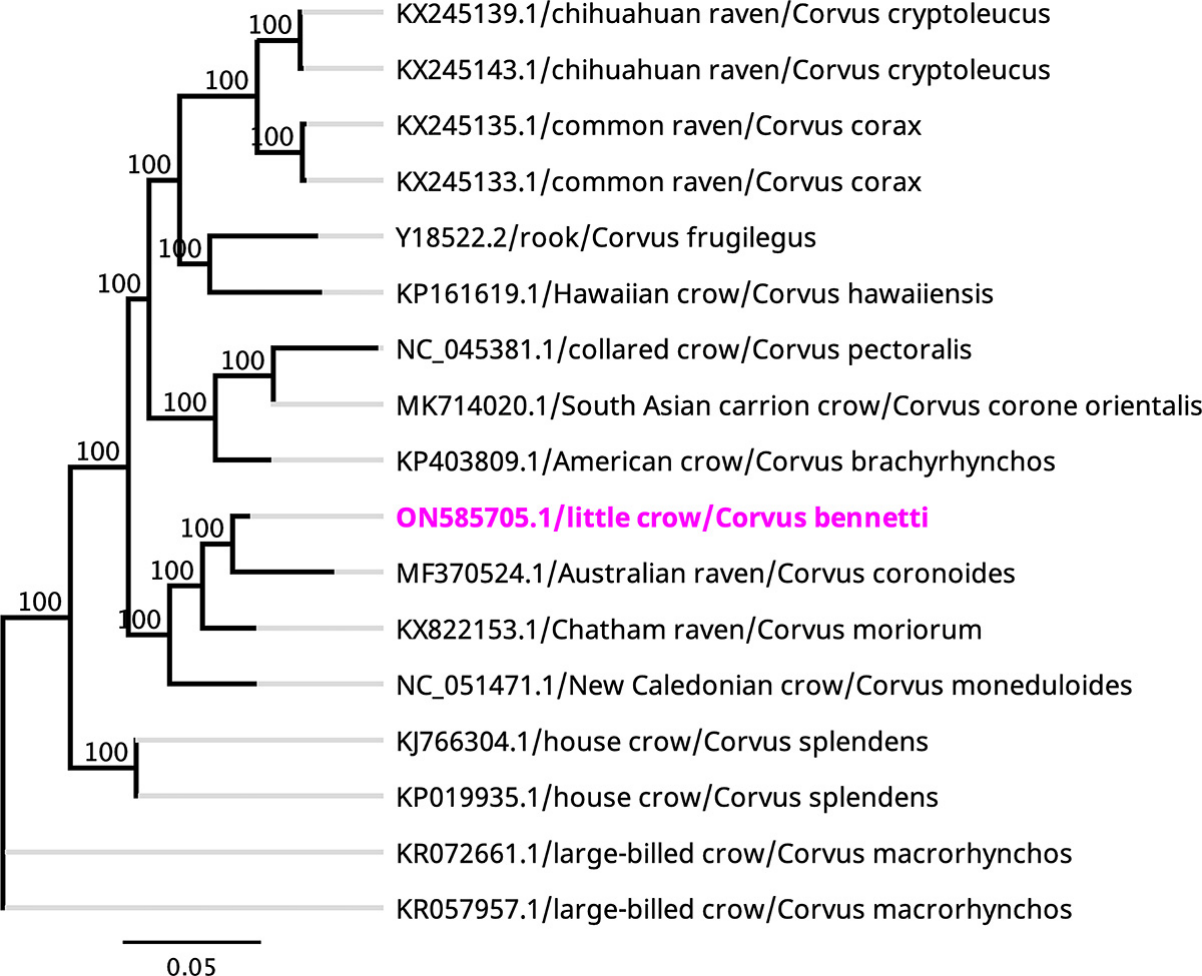
Maximum likelihood phylogenetic tree to infer host phylogenetic relationships using the entire mitochondrial genome sequenced from *C. bennetti*, along with those of other selected species within the order Passeriformes. The nucleotide sequences were aligned using MAFFT version 7.450 with the G-INS-i algorithm (gap open penalty, 1.53; offset value, 0.123) implemented in Geneious Prime version 7.388 (12). The phylogenetic tree was determined using the general time-reversible (GTR) substitution model with 500 bootstrap replicates using Geneious Prime. The new complete mitogenome of *C. bennetti* is shown in magenta. Labels at the branch tips indicate the GenBank accession number, followed by the common name and organism.

The assembled complete little crow mitogenome has a circular genome of 16,895 bp, with a G+C content of 44.4%. The annotated mitogenome contains two rRNA-encoding regions (12S and 16S rRNA), 22 tRNAs, and 13 protein-coding genes (PCGs). Phylogenetic analysis using the complete mitogenome sequences showed that *C. bennetti* grouped into a well-supported subclade with the Australian raven (Corvus coronoides; GenBank accession number MF370524.1) (Fig. 1) (11), with which it demonstrated the highest pairwise nucleotide identity (96.23%). The complete mitogenome of *C. bennetti* provided by this study will be a useful reference for the family *Corvidae* to further study host phylogenetic relationships.

### Data availability.

The complete mitochondrial genome sequence of *C. bennetti* has been deposited at DDBJ/ENA/GenBank under the accession number ON585705. The version described in this paper is the first version, ON585705.1. The raw sequencing data from this study have been deposited in the NCBI Sequence Read Archive (SRA) under the accession number SRR19117728 (BioProject accession number PRJNA835616).
